# Macon Medical Society

**Published:** 1884-10

**Authors:** 


### Society Reports.

### MACON MEDICAL SOCIETY.

Reported for THE ATLANTA MEDICAL AND SURGICAL JOURNAL.

STATED MEETING, September 2d, 1884.

Dr. C. H. Hall, president, in the chair,

Dr. H. McHatton read the introductory essay, subject:

*Examination of a Specimen of Urine from a Case of Malarial Hæmaturia.*—The specimen was brownish black, corresponding to No. 9 of Meibauer and Vogel's color plates; odor slightly urinous; reaction faintly acid. Specific gravity 1012, albumen about 95 per cent. of bulk.

Microscopical examination revealed a few epithelial casts; three or four to the slide; crystals of ox. of lime and a considerable amount of brownish amorphic matter, with the regular train of unimportant constituents. Although the urine was fresh, on repeated examination I could not find a single red or white blood corpuscle. The specimen was then filtered. One part of the filtered urine was treated with a little sodie hydrate, heated to boiling and allowed to stand—the precipitate was brownish red, dichrotic, playing into green by reflected light, showing the presence of the blood constituents, but not allowing a discrimination between hæmatin, hæmoglobin and methannoglobin.

A second part was treated with sodie hydrate, then with a solution of tannin, and lastly with acetic acid, which gave the brownish precipitate of tannate of hæmatin; this precipitate was washed and dried, treated with chloride of sodium and glacial acetic acid, then boiled on a slide, giving as a result an immense number of the characteristic hæmin crystals.

A third part was treated with an emulsion of equal parts of tr. guiac and oil of turpentine, giving a most intense indigo blue reaction.

A specimen of the vomit from the same patient gave hæmin crystals.

This examination proved most conclusively that whereas the urine contained none of the anatomical elements of the blood it was loaded with the chemical constituents. In fact, it was a case of hæmoglobinuria and not hæmaturia, at the same time showing little or no renal trouble.

The question naturally rose whether the entire mass of the blood was not decomposed (more or less) into its chemical constituents.

Poufick's experiments on transfusion throw an unexpected light on the etiology of hæmoglobinuria, as they prove that it is due to a breaking down of the red blood corpuscles in the body at large, and not to any kidney affection. This pathological state of the blood occurs in scorbutus, in the morbus maculosa of Bischoff, in hemorrhagic small pox; in typhus-septicæmia and poisoning by phosphorus, arsenic, sulphuretted hydrogen, etc., to which, from my own observation, I may add, chlorate of potash. Vogel divides these cases into two classes:

1. Where the cause is a temporary one, in which the evil results are confined to the loss of a greater or less quantity of blood corpuscles, and the prognosis is favorable—cases of mild poisoning.

2. Where the cause operates permanently, the prognosis is unfavorable. For example scurvy, if left to nature, malarial hæmaturia comes under this head. If we accept the etiology of malarial hæmaturia that its name indicates, and look upon the disintegration of the blood corpuscles as dependent on a profound malarial toxemia, quinine must be our sheet anchor. We should begin its use on the first show of the constituents of the blood in the urine, and push it to cinchonism.

I should advise the use of the following test, which is simplicity itself, whenever the color of the urine becomes the least suspicious:

"Mix an equal part of tincture of guiac. and oil of turpentine. Shake till an emulsion is formed. When the urine comes in contact with the emulsion the guiac resin is quickly precipitated, as a white, later, dirty yellow or green precipitate. If the urine contains blood, or in traces merely, the resin is colored more or less intensely blue, often almost indigo blue (almin)."

If this test shows hæmoglobinuria I should advise the immediate administration of at least thirty grains of the sulphate of quinia, and if at the end of three hours there are no evidences of cinchonism, repeat the same dose. When a moderate degree of cinchonism is produced, it should be kept up by smaller doses of quinine, until the danger of the next paroxysm has passed. If the quinine is not tolerated by the stomach, it should be given hypodermically.

During this time the patient must be treated symptomatically.

Support the heart's action by its appropriate stimulants. Coldness of surface and extremities calls for the external application of heat. Morphia is indicated if there is much restlessnes and delirium.

Cathartics, on account of their depressing effect, seem to me to be decidedly contra-indicated.

In convalescence the preparations of iron and small doses of the bichloride of mercury are indicated for their blood-producing properties.

Good hygienic surroundings should be insisted upon, and a temporary change of climate procured if possible.

Dr. J. P. Stevens said:

MR. PRESIDENT: I have been much pleased and entertained by Dr. McHatton's essay, especially as the results of his investigations confirm my preconceived ideas in relation to the pathological condition of the blood, as well as the tissues in hemorrhagic malarial fever. The breaking down of the red and white corpuscles, it appears to me, may be accounted for upon the principle of animal fermentation induced by microbes in the blood.

During the last visitation of New Orleans by yellow fever, Dr. Schmidt discovered a fatty degeneration of the muscular tissue, as well as of the glandular textures of the abdominal secretory organs and of the tissues of the heart. The dermoid tissue seemed to be remarkably free from this degenerative change. He advanced the idea that if this metamorphic degeneration continued until the plastic or nitrogenized elements of nutrition became involved, death would be the inevitable result. Any process short of this might, under favorable conditions, be arrested until the recuperative powers of nature should restore a normal condition of the tissues.

Now, it would seem from the pathological phenomena revealed and detailed in the essay just read, as well as from the symptoms observed in the progress of the disease, that analogous tissue change may be present in the subject of hemorrhagic malarial fever. The black vomit of this disease we believe to be identical with that of yellow fever. In the latter this phenomenon arises from a rupture of the submucous venules, and by a process of transudation the blood passes into the stomach and is thence ejected. So it is in hemorrhagic malarial fever. By this transudation process the so-called intestinal and renal hemorrhages may be accounted for. The functions of the liver and kidneys seem to be in a considerable measure impaired, and in the latter stages of fatal cases completely arrested.

Having treated the disease over a period of ten years, in a portion of the State where it is most prevalent, and having been a subject of it myself, my opportunities for becoming acquainted with its external manifestations are rather exceptional. Its type is evidently asthenic, as we would infer from the destructive degeneration of the blood corpuscles. The oxygen-carrying function of the red corpuscles as well as the alimentation function of the white corpuscles, must necessarily be seriously crippled. I have never known a case of this disease that was not preceded by frequently recurring attacks of intermittent fever; and with those who have been habituated to the excessive use of alcoholic stimulants in health, the disease as a rule is fatal. The excessive prostration of the vital powers, weak heart action, distressing sleeplessness, almost bronzed hue of the skin, paucity and perversion of the hepatic, renal, gastric and pancreatic secretions, as well as the impoverishment of the nerve tissue, all indicate an overwhelming depression occasioned by blood poisoning, or you may say by the depredations of the *bacillus malariæ.*

Now, if the pathology assumed be correct, what are the indications for treatment? Evidently the sustaining and tonic plan. By judicious medication, keeping the cutaneous emunctories in as good condition as possible, by administering as much simple nutriment as the stomach can bear, and by alcoholic stimulation. A combination of muriate of quinine, or the sulphate—I prefer the former—chlorate of potash, and tr. mur. ferri. in solution, I have found to be followed by the best results. And my success has been exceptional in accomplishing good results. This combination should not be given in heroic doses, but so as to get the full benefit of their sustaining power by repeating at intervals of two or three hours until the system becomes cinchonized.

One of the most dreadful symptoms we have to contend with is the insufferable nausea that torments the patient night and day. Large doses of quinine, 20 or 30 grs., irritate the stomach, and are usually speedily ejected. Moreover, where there is such a continual drain from the excessive renal and intestinal discharges, the antipyretic properties of this drug are not imperatively demanded. We often commit a grave error by over-dosing. I was informed of an estimable gentleman who was said to have been drugged by his physicians with an ounce of quinine during a few days' illness. Of course he died. In the combination before mentioned, the chlorate of potash seems to act beneficially, but precisely in what manner I am not prepared to say; probably in some beneficent way upon the renal secretion; we know that it cannot impart its oxygenizing properties to the blood, as it does not part with its oxygen at the temperature of the body.

Affusions of water at a temperature most congenial to the feelings of the patient should be sedulously observed. The eliminating power of the cutaneous secretion is greatly promoted by this measure, and it adds very much to the comfort of the patient. Excessive nausea may be relieved by the subcutaneous injections of morphia, and if inveterate, an epispastic should be applied to the epigastrium, and allowed to remain just long enough to slightly vesicate the surface.

Proper alimentation can be best accomplished by the use of peptonized or pancreated milk. The function of the gastric glands is kept in abeyance; hence the deficiency if not the complete arrest of the gastric juice. We must, therefore, aid the feeble assimilative powers by the partial artificial digestion of the milk before it is taken into the stomach. Whisky or good brandy may be administered as freely as the stomach will bear, in quantities indicated by the necessities of the patient.

We must ever bear in mind that the pabulum of life is poisoned and disintegrated, and the vital powers are overwhelmed by a fearful incubus; that we can not cure, but must sustain, strengthen and comfort the patient, until nature is enabled to lift up the burden, and by her own methods restore the equilibrium of the forces.

Dr. C. H. Hall said he would not recommend such large doses of quinine unless in the first stages, as the depressing effect could not be overcome by stimulants. Quinine was indicated if the patient was not seen till after the disease had made much progress; would advise 3 to 4 grain doses; earlier in the case would give as high as fifteen.

Dr. McHatton said: Wishing to be as brief as possible in my paper, I of course did not go into details. Referring to it I find: "We should begin its use (quinine) on the first show of the constituents of the blood in the urine," which is followed by as far as

I know the most delicate bedside test for said constituents. That would be before the patient was depressed, and in the majority of cases earlier than the disease would usually be diagnosed.

I do not advise these doses of quinine for their antipyretic effect, but their controlling effect on the malarial poison and through that action to stop the disintegration of the red blood corpuscles.

With Dr. Stevens I am of the opinion that a *post mortem* on one of these cases would reveal a state of malanæmia; in malarial malanæmia quinine is the recognized remedy, and it presumably acts by removing the cause. Victims of the alcoholic habit should of course follow the general rule in this disease and succumb easily.

I did not go through the list of stimulants. The alcoholics are my favorites, and I give them hypodermically when there is any danger of their being ejected by the stomach.

I do not wish to take issue with Dr. Stevens on his clinical experience with chlorate of potash, still I have seen hæmoglobumin produced by large doses of it, and cannot see the *rationale* of its use in these cases. I call his attention to the fact that he used it in conjunction with sufficient quinine to produce cinchonism.

Since writing my paper, I see that Drs. Machifara and Celli have succeeded in demonstrating that the germs of a certain schizomycate attack directly the red blood corpuscles, causing them to undergo a series of very characteristic changes which admit of easy verification and which render certain the existence of a malarial infection.

---

DOCTORS MUST NOT TELL.—The Missouri Supreme Court has decided that information obtained by a physician from a patient must not be disclosed on the witness stand, though the physician declares that the information was acquired while in a professional capacity, and was necessary to enable him to prescribe as a physician or operate as a surgeon. The court held that it would not do, while the mouth of a physician is closed as to the talk of a patient, to open it as to knowledge acquired from his own diagnosis of the case.

